# Treatment Protocols for Gestational and Congenital Toxoplasmosis: A Systematic Review and Meta-Analysis

**DOI:** 10.3390/microorganisms13040723

**Published:** 2025-03-24

**Authors:** Sissi Kelly Ribeiro, Igor Moraes Mariano, Ana Clara Ribeiro Cunha, Ana Cláudia Arantes Marquez Pajuaba, Tiago Wilson Patriarca Mineo, José Roberto Mineo

**Affiliations:** 1 Laboratory of Immunoparasitology, Institute of Biomedical Sciences, Federal University of Uberlândia, Uberlândia 38400-678, MG, Brazil; sissi.ribeiro@ufu.br (S.K.R.); ana.pajuaba@ufu.br (A.C.A.M.P.); tiago.mineo@ufu.br (T.W.P.M.); 2 Laboratory of Cardiorespiratory and Metabolic Physiology, Federal University of Uberlândia, Uberlândia 38405-302, MG, Brazil; igor.mariano@ufu.br (I.M.M.); anacrcunha@ufu.br (A.C.R.C.)

**Keywords:** congenital toxoplasmosis, maternal and newborn treatment, efficacy, therapeutic failure, diagnostics, sequelae

## Abstract

Toxoplasmosis is a globally prevalent zoonotic parasitic disease. Neonates with congenital infection can develop severe long-term sequelae, which can be mitigated or prevented through early diagnosis and therapeutic approaches. In this context, the main objective of this study was to describe the main treatments and evaluate the effectiveness of the current treatment protocols for gestational and congenital toxoplasmosis to prevent vertical transmission and to reduce clinical manifestations in neonates. This systematic review with a meta-analysis searched digital databases (PUBMED, SCOPUS, WEB OF SCIENCE, EMBASE, and COCHRANE) for observational cohort studies published between 1 January 2013 and 29 January 2025, evaluating treatment effectiveness in gestational and congenital toxoplasmosis. Risk ratios (RRs) were calculated using random effects models to assess infection risk and clinical manifestations in neonates. The study quality was assessed following the Joanna Briggs Institute protocol and fifty-six studies from 16 countries were included, comprising 11,090 pregnant women and 4138 children. Studies were predominantly from Brazil (38%), France, and Italy. Only 9% of the studies indicated knowledge of the serological status of the pregnant woman before the gestational stage. Of 10,148 women with confirmed toxoplasmosis, 8600 received treatment, with 18% of their children infected, compared to a 58% infection rate in untreated mothers’ children. Meta-analysis showed that treatment reduced infection risk (RR = 0.34 [0.21; 0.57]) and clinical manifestations (RR = 0.30 [0.17; 0.56]). While spiramycin or triple therapy showed similar effects, triple therapy demonstrated more consistent results (RR: 0.22 [0.15; 0.32]) compared to spiramycin alone (RR: 0.54 [0.06; 4.67]). In conclusion, treatment protocols for congenital or gestational toxoplasmosis have proven to be effective in reducing the risk of infection and clinical manifestations in neonates. Regarding the type of treatment, although they have similar responses, the use of triple therapy shows more consistent responses than isolated spiramycin. It can be also concluded that prevention and mitigation of congenital toxoplasmosis require standardized treatment protocols, improved diagnostic methods, and educational programs for women of childbearing age, as treatment initiation timing and protocol choice are crucial factors in determining outcomes.

## 1. Introduction

*Toxoplasma gondii* is one of the most successful parasitic pathogens worldwide, capable of infecting a remarkable range of avian and mammalian hosts, including humans [1]. This protozoan’s heteroxenous life cycle, with felids as definitive hosts, contributes to its widespread distribution and significant public health impact [2,3]. Humans can be infected by several routes, by eating undercooked meat of animals containing tissue cysts, by consuming food or water contaminated with cat feces, by contaminated environmental samples, by blood transfusion or organ transplantation, as well as transplacentally from mother to fetus [1]. While typically asymptomatic in immunocompetent hosts, *T. gondii* infection poses significant health challenges in two critical scenarios: immunocompromised individuals, where it can cause severe neurological complications, and pregnant women, where transplacental transmission puts the fetus at risk of congenital infection [3]. In addition to these implications, socioeconomic factors such as lower socioeconomic status and education of the population play a significant role in the prevalence of toxoplasmosis. Although socioeconomic factors influence toxoplasmosis prevalence, congenital infection can affect all demographic groups, making it a universal concern in maternal healthcare [4]. 

Considering this complex scenario, the therapeutic approach to toxoplasmosis is a significant challenge [5]. Conventional treatment is limited to the acute phase of the disease, with no effects on latent parasites; consequently, a cure is still not available. Furthermore, considerable toxic effects and long-term therapy contribute to high rates of treatment abandonment [6]. However, evidence from French studies suggests that preventive and therapeutic approaches during pregnancy can reduce the risk of symptoms and sequelae in children [7].

Regarding the type of treatment, anti-*Toxoplasma* chemotherapy consists of several medications that can be used individually or in combination. These include sulfadiazine (SDZ), pyrimethamine (PYR), sulfamethoxazole (SMT), trimethoprim (TMP), and spiramycin [8]. Currently, the gold standard treatment involves a combination of SDZ and PYR, with the option to use the combination of SMT and TMP, clindamycin, and spiramycin, all of which present a synergistic effect affecting the replication of the tachyzoite form of *T. gondii* [9,10]. New research on the impacts of toxoplasmosis highlights the need to increase institutional awareness of infection pathways and implement comprehensive, interdisciplinary actions to control transmission and optimize treatment [11].

Given the complex clinical context and varied therapeutic approaches, this systematic review with a meta-analysis aims to evaluate treatment protocols for gestational and congenital toxoplasmosis, focusing on their effectiveness in preventing vertical transmission and reducing clinical manifestations in newborns.

## 2. Methods

### 2.1. Search Strategy and Selection Criteria

This systematic review and meta-analysis was conducted following the Preferred Reporting Items for Systematic Reviews and Meta-Analysis (PRISMA-P) guidelines [12], and the method protocol used was previously published (Available online: https://www.protocols.io/view/protocol-of-a-systematic-review-with-metanalysis-f-e6nvwddp2lmk/v1 accessed on 10 February 2025) and registered on the PROSPERO platform (Available online: https://protocols.io/view/protocol-of-a-systematic-review-with-metanalysis-f-cymnxu5e.pdf accessed on 10 February 2025).

Searches were conducted in PUBMED, SCOPUS, WEB OF SCIENCE, EMBASE, and the COCHRANE Library, manually via Google Scholar, plus with reference lists of key articles, for studies published between 1 January 2013 and 29 January 2025. The search strategy combined three categories of terms (Treatment, Congenital toxoplasmosis, and Population) using Boolean operators ‘OR’ within categories and ‘AND’ between categories, as follows: (Treatment OR Drug OR Drugs OR Spiramycin OR Sulfadiazine OR “Folinic acid” OR Pyrimethamine) and (“Congenital toxoplasmosis”) and (Maternal OR Pregnant OR Children OR Child OR Newborn OR Baby OR Babies OR Pediatric OR Infant OR Neonate).

Studies were eligible with the following characteristics: (1) population: human, pregnant, and newborn; (2) intervention: treatment of toxoplasmosis; (3) control/comparator: children/mothers not treated for toxoplasmosis; (4) outcomes of interest: treatment protocols used and their effectiveness in reducing risk of vertical transmission and clinical manifestations in neonates; (5) languages: there was no language restriction; while no design restrictions were applied, only cohort studies (prospective or retrospective) met the inclusion criteria of reporting both *T. gondii* infection status and treatment data; (6) publication dates: within the specific time frame that has been set.

Excluded works included literature reviews, meta-analyses, letters to the editor, animal studies, studies unrelated to toxoplasmosis and treatment, studies that treat other comorbidities in addition to toxoplasmosis, full texts that were not accessible, and texts published before 2013. To avoid bias in the results, studies whose mothers had co-infection were excluded.

Regarding outcome assessment, vertical transmission was confirmed through serological tests (IgM and IgG), PCR in amniotic fluid, or placental examination. Clinical manifestations were documented via ophthalmological examination, neurological assessment, and imaging studies. Treatment effectiveness was evaluated by comparing infection rates and clinical manifestations between treated and untreated groups. These standardized outcome measures were used to ensure consistency in data extraction and analysis across included studies.

First, the titles and abstracts of the research studies were evaluated independently by two researchers. Duplicates and studies that did not meet the inclusion or exclusion criteria were excluded from the analyses. Title and abstract screening were conducted using Rayyan (https://www.rayyan.ai/), accessed on 10 February 2025, a software for organizing and managing systematic reviews independently, where each reviewer performed article selection blindly [13]. During the selection process, all data were cross-checked, and discrepancies were resolved through consensus and, if necessary, by the senior researcher. Finally, the full texts of eligible articles were read by the same researchers to decide on their definitive inclusion. For articles considered relevant to the review but not available in full for reading, authors were contacted to request the availability of the full article.

Data were collected using a standardized Excel spreadsheet (Microsoft Excel^®^, version 2016). The data extraction included the following: (1) bibliometrics (country where the study was conducted, title, journal, language, DOI, and publication year); (2) time of diagnosis; (3) initiation phase of therapy, therapeutic options; (4) dosage form of drugs; (5) dosage; (6) main clinical manifestations and sequels; (7) duration of treatment; (8) patient follow-up; (9) number of treated and untreated patients; (10) idiosyncrasies of the population or treatment. 

After the selection phase, the data were extracted, and the quality assessment was performed using the Joanna Briggs Institute (JBI) critical appraisal checklist for cohort studies, which evaluates key methodological aspects including population selection, exposure measurement, identification of confounding factors, outcome assessment, and follow-up adequacy. Studies were rated on each criterion as ‘yes’, ‘no’, ‘unclear’, or ‘not applicable’ [14].

### 2.2. Data Synthesis and Analysis

The data were evaluated using the programming language “R” [15] through the supplements “meta” [16] and “metafor” [17]. The pooled effect estimates were computed from risk ratio (RR) differences between treated and untreated groups using random effects models due to expected clinical and methodological heterogeneity between studies. Statistical heterogeneity among studies was evaluated by Cochran’s Q test and *I*^2^ inconsistency test [18]. Data synthesis involved pooling RR for infection and clinical manifestations separately, with subgroup analyses performed by treatment type. Subgroups analysis was conducted through comparing the incidence of cases of congenital toxoplasmosis after different treatments in pregnant women. Forest plots were generated to present the pooled effect and the 95% confidence interval. 

## 3. Results

### 3.1. Qualitative Results

**Figure 1:**
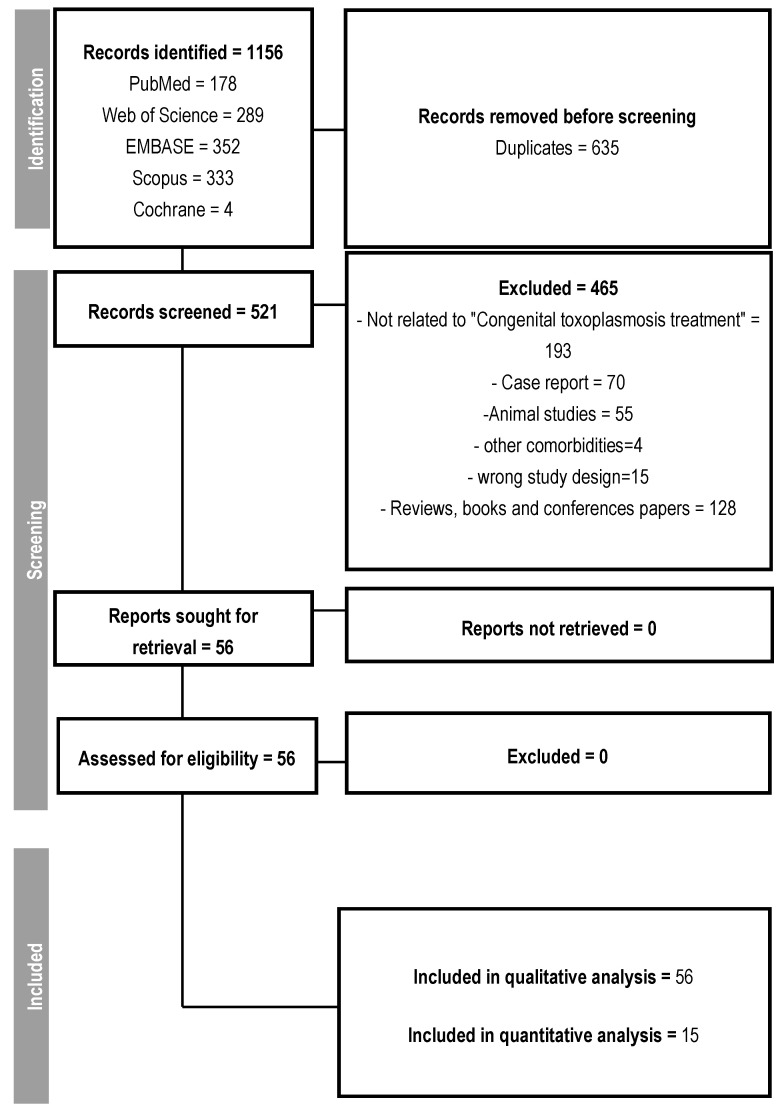
Follow-up flowchart.

From the initial selection of 1156 studies, 635 were duplicates. Out of the remaining 521, 465 were excluded for pre-defined reasons, such as studies unrelated to the treatment of congenital toxoplasmosis, case reports, studies conducted on animals, reviews, and presentations at scientific conferences, resulting in the selection of 56 studies that addressed the treatment of congenital or gestational toxoplasmosis (Figure 1). No article was excluded due to its inaccessibility.

All 56 eligible studies contributed to the construction of data to elucidate the evidence, considering the following variables: time of diagnosis; initial phase of therapy, therapeutic options; pharmaceutical form of medications; dosage; main clinical manifestations and sequelae; treatment duration; patient follow-up; number of treated and untreated patients; population or treatment idiosyncrasies. The characteristics of all studies included in the qualitative analysis are shown in Supplementary Table S1. A total of 11,090 pregnant women and 4138 children were enrolled in these studies. Eligible studies were published between 2013 and 2025 in 16 countries. Out of the total number of studies analyzed, 20 were conducted in Brazil, followed by France and Italy, with 6 studies each. Moreover, 85% of the studies were conducted in Referral Centers.

Some studies have shown that the screening period for toxoplasmosis during pregnancy may vary from country to country, but indirect diagnostic methods for detecting IgM and IgG immunoglobulins were unanimous, albeit with variations in serological methodology [19,20,21,22]. In this regard, one of the mentioned diagnostic methods was the Sabin Feldman test, considered the gold standard for toxoplasmosis diagnosis. Some studies have demonstrated the use of other immunoglobulins such as IgA and IgE [23,24,25,26,27,28,29,30,31,32] to complement the diagnosis of *Toxoplasma* infection. Another methodology addressed in the studies was the avidity test, considered a useful tool for detecting the timing of infection [33]. Ultrasound imaging examinations in pregnant women were also present in all studies, while the PCR technique was present in 50% of the studies.

Regarding the knowledge of the pregnant woman’s serological status before the gestational stage, a relevant factor in defining the timing of infection if the infection was acquired during or early in pregnancy, which would facilitate the detection of the moment of seroconversion. However, this analysis was addressed in only 9% of the studies.

A total of 2652 IgG avidity tests were conducted, with 1595 of them detecting low avidity. Additionally, a total of 4284 PCR tests were performed, with 3724 on amniotic fluid and 560 on placenta or umbilical cord, resulting in 442 positive results for a total of 995 children diagnosed with congenital toxoplasmosis with follow-up after one year of age. Additionally, routine follow-up tests for pregnant women considered seronegative for toxoplasmosis during prenatal care are monthly in France and Italy [3,28,31,34,35,36]. Ultrasounds were conducted on pregnant women as a complementary examination to indirect diagnostic tests, and in children, fundoscopic eye examinations, transfontanelle ultrasounds performed at birth, and physical and neurological examinations were carried out in all studies conducted at reference centers. 

According to Damar et al. [20], in Turkey, specifically in the city of Sanlúrfa, there is no established protocol considering that it is not a highly prevalent disease in the country. Similarly, in Japan, the frequency of prenatal screening exams, according to Hijikata et al. [37], is quarterly since it does not have a high incidence. In Vienna, Austria, the frequency of exams is bimonthly [22]. According to Carral [19], in Buenos Aires, Argentina, exams are repeated every trimester and during childbirth, and in Brazil, exams are repeated quarterly [38,39,40]. 

Out of a total of 10,148 pregnant women diagnosed with toxoplasmosis, 8600 received some form of treatment and had 1548 (18%) infected children, while 1586 untreated mothers had 922 children infected with *T. gondii* (58%).

In 71% of the studies, the triple therapy regimen (sulfadiazine, pyrimethamine, and folinic acid) associated or not with spiramycin is used as a treatment for gestational and congenital toxoplasmosis, with the following dosage regimens: pyrimethamine 25–50 mg/day, sulfadiazine 3 g/day, folinic acid 25–50 mg twice a week, and spiramycin 3 g/day. For neonates, the established regimen is pyrimethamine 3 mg/kg every 3 days, sulfadiazine 25 mg/kg every 8 hours, and folinic acid 50 mg every 7 days orally [41].

Only five studies cited the pharmaceutical form adopted for the treatment of neonates/children. The most common clinical manifestations in children of treated mothers were ophthalmological, while in children of untreated mothers, the predominant clinical manifestations were neurological. In total, 113 deaths were reported, of which 48% were spontaneous abortions, and 25% were terminations of pregnancy after amniocentesis results [26,42,43], with the remainder being represented by 15% stillbirths, 11% postnatal deaths, and 1% death during adolescence. 

### 3.2. Quantitative Results

To perform the meta-analysis, only studies with groups larger than 10 individuals per group and that did not include only infected individuals were considered. This approach reduces the risk of bias associated with small sample sizes and studies in which both groups would have all individuals infected, which would not allow for infection risk analyses. As shown in Table 1, a total of 15 studies were included in the meta-analysis, some of which contained infection risk data, while others presented clinical manifestation data, leaving 10 for each analysis. However, the timing of maternal infection, a potential important confounder, could not be adequately analyzed as this information was not consistently reported across studies. Although the exact timing of infection was uncertain, studies reported mean treatment initiation at 24 ± 6 gestational weeks.

**Figure 2:**
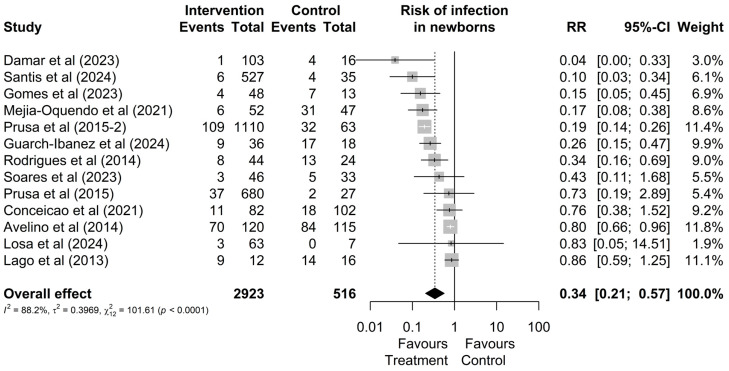
Forest graph of the risk of infection in the fetus after gestational treatment for toxoplasmosis [20,22,24,30,38,44,45,46,47,48,49,50,51].

Regarding the risk of infection, out of the 2923 mothers treated during pregnancy and the 516 who remained untreated, it was observed that a total of 276 (9%) and 231 (45%) had infected newborns, respectively (Figure 2). Therefore, treatment reduces the possibility of vertical transmission (RR = 0.34 [0.21; 0.57]; Figure 2). Additionally, no outlier values were identified regarding the risk of infection.

**Figure 3:**
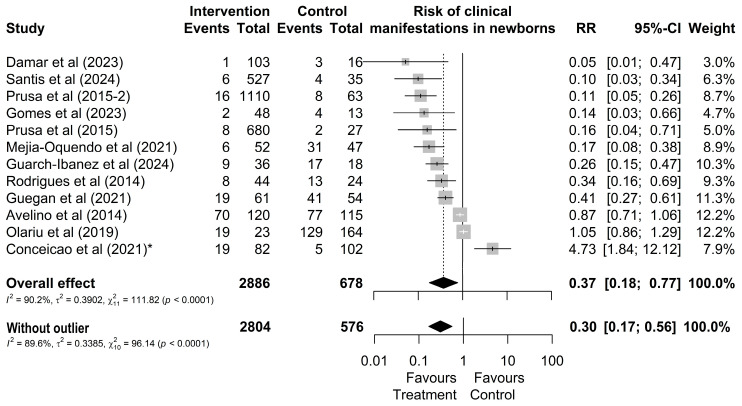
Forest chart of the risk of clinical manifestations in children after gestational treatment for toxoplasmosis. (*) The outlier study [20,21,22,24,27,38,44,45,46,49,50,51].

Regarding the clinical manifestations of newborns from treated or untreated mothers, it was observed that out of 2886 treated mothers, 183 (6%) had children who presented some type of clinical manifestation (Figure 3). In contrast, out of 678 untreated mothers, a total of 334 (49%) of their children presented one of the typical clinical manifestations (Figure 3). However, the study by Conceição et al. [24] was identified as an outlier and influential point without overlapping effects with other studies. By omitting this study, the result changes from RR = 0.37 [0.18; 0.77] (Figure 3) to RR = 0.30 [0.17; 0.56].

### 3.3. Comparative Analysis Between Treatments

**Figure 4:**
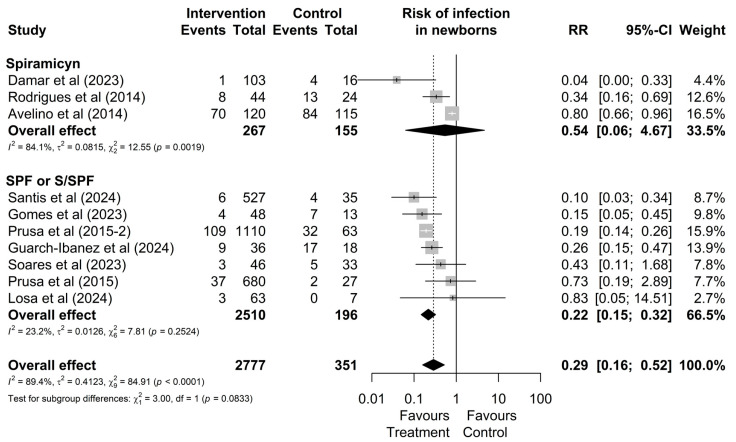
Forest plot of the proportion of infected neonates divided into treatment drug subgroups. CI: confidence interval; studies with more than one drug subgroup; SPF: sulfadiazine + pyrimethamine + folinic acid; W/SPF: spiramycin alternated with SPF [20,22,30,38,44,45,46,48,50,51].

As shown in Figure 4, although no significant difference between treatments was observed in the overall analysis (*p* = 0.0833), the subgroup of studies involving mothers treated with the triple regimen demonstrated lower heterogeneity (*I*^2^ = 23.2%, χ^2^ *p* = 0.18) and statistically significant results (RR: 0.22 [0.15; 0.32]) compared to the group treated with spiramycin alone (*I*^2^ = 84.1%, χ^2^ *p* < 0.01), which showed non-significant results (RR: 0.54; [0.06; 4.67]). These findings suggest greater consistency in treatment effects for mothers who received the triple regimen.

### 3.4. Risk of Bias

**Figure 5:**
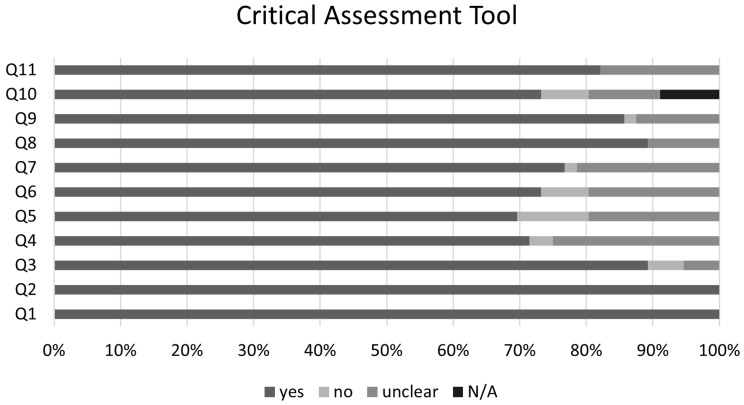
Joanna Briggs Institute Critical Assessment Tool. Q1. Were the two groups similar and recruited from the same population? Q2. Were the exposures measured similarly to assign people to both exposed and unexposed groups? Q3. Was the exposure measured in a valid and reliable way? Q4. Were confounding factors identified? Q5. Were strategies to deal with confounding factors stated? Q6. Were the groups/participants free of the outcome at the start of the study (or at the moment of exposure)? Q7. Were the outcomes measured in a valid and reliable way? Q8. Was the follow up time reported and sufficient to be long enough for outcomes to occur? Q9. Was follow up complete, and if not, were the reasons to loss to follow up described and explored? Q10.Were strategies to address incomplete follow up utilized? Q11. Was appropriate statistical analysis used? N/A—not applicable.

**Figure 6:**
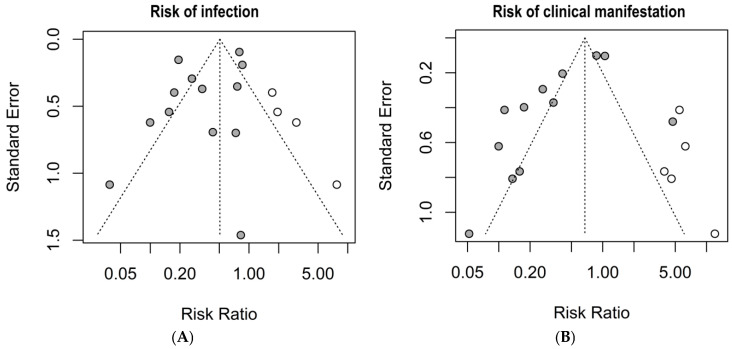
Publication bias risk by the trim-and-fill method and funnel plots. (**A**) risk of infection; (**B**) risk of clinical manifestations. Grey circles: Published studies included in the meta-analysis. White circles: Hypothetical missing studies identified through trim-and-fill analysis. These represent potentially unpublished studies that, if included, would balance the funnel plot and produce a more symmetrical distribution around the estimated true effect size.

The graphical summary of the bias risk assessment using the Joanna Briggs Institute critical appraisal tool for all studies included in qualitative and quantitative analyses is shown in Figure 5. Overall, the analyzed studies demonstrated robust methodological quality, with 84% average adherence to the assessment instrument criteria. The risk of bias evaluation revealed that most studies (81%) presented a low risk, while only 11% showed moderate risk, and 8% were classified as high risk. This favorable distribution of bias risk levels substantiates the methodological rigor of the included studies, enhancing the reliability of the findings. No authors reported conflicts of interest. Regarding publication bias, the Egger’s test did not show an increased risk; however, funnel plots with the trim-and-fill method suggest the possible omission of three studies regarding the risk of infection and four studies regarding the risk of clinical manifestations (Figure 6).

## 4. Discussion

The most effective approach to preventing congenital toxoplasmosis requires comprehensive management from diagnosis through treatment of the mother–child pair. Our meta-analysis demonstrated that maternal treatment significantly reduces both vertical transmission risk (RR = 0.34 [0.21; 0.57) and clinical manifestations in newborns (RR = 0.30 [0.17; 0.56]). While both spiramycin alone and triple therapy (sulfadiazine, pyrimethamine, and folinic acid) showed similar efficacy, triple therapy demonstrated more consistent results with lower heterogeneity between studies. In this systematic review, only 9% of studies reported prior knowledge of women’s serological status during the preconception period, highlighting a critical gap in preventive care. These findings emphasize the urgent need to implement protocols promoting early serological screening in women of childbearing age, particularly in countries with high toxoplasmosis incidence, as determining the timing of infection is crucial for optimal therapeutic management [36].

The implementation of monthly serological monitoring represents a critical strategy in preventing congenital toxoplasmosis. This is particularly evident when examining data from certain regions of high-incidence countries like Italy [28] and Brazil [38], where reported rates are 0.12% and 0.15%, respectively. While economic constraints in low- and middle-income countries often challenge the feasibility of monthly testing during prenatal care, the scientific evidence strongly supports that regular monitoring is essential for early detection and appropriate intervention [32]. This is primarily because the effectiveness of antiparasitic treatment significantly decreases once the parasite establishes intracellular infection beyond the initial parasitemia phase.

The critical importance of comprehensive diagnostic approaches is further emphasized by discordant immunoglobulin levels as reported by Fricker-Hidalgo et al. [34], demonstrating that interpretation of current tests extends beyond standard parameters. Misinterpretation or relativization of results outside recommended levels may result in irreversible fetal harm. Beyond standard IgG and IgM measurements, complementary testing for anti-*Toxoplasma* IgA and IgE isotypes [27,29], PCR techniques [36], and proper timing of diagnostic procedures are crucial for accurate diagnosis. The PCR test timing is particularly critical, as it should be performed between the 16th and 30th weeks of gestation and not exceed 4–6 weeks from the estimated infection date to avoid false-negative results. However, the accuracy of the PCR method remains unclear, as the occurrence of true false positive and false negative rates still need to be determined, even considering that diagnosis of congenital toxoplasmosis using a combination of IgG avidity in maternal blood and multiplex nested PCR in amniotic fluid and neonatal blood is helpful to detect a high-risk pregnancy, as well as to diagnose *T. gondii* infection [32].

The number of infected cells and intracellular parasite concentration significantly influence infection pathophysiology, emphasizing the importance of early and accurate diagnosis for optimal prognosis [50]. Variations in PCR diagnostic outcomes may result from multiple factors, including reduced test sensitivity, suboptimal timing of specimen collection relative to infection onset [29,34,50], or treatment initiation effects [27]. These technical challenges underscore why a comprehensive diagnostic approach is essential, incorporating multiple methodologies to ensure accurate detection and monitoring.

While this multi-method diagnostic approach represents the ideal standard of care, its implementation faces significant challenges, particularly in resource-limited settings. Healthcare systems in low- and middle-income countries must balance the substantial costs of comprehensive testing against the potential consequences of delayed or missed diagnoses. This economic consideration becomes particularly relevant given that regular monitoring remains fundamental for successful treatment outcomes, as the parasite’s intracellular behavior after initial parasitemia significantly reduces antiparasitic drug efficacy.

The complexity is further compounded by the specificity of acquired immunity to primary *T. gondii* genotype contact. Exposure to different genotypes requires development of new immunological memory, highlighting the importance of continued serological monitoring throughout pregnancy to identify recent infections. This biological characteristic, combined with the time-sensitive nature of effective intervention, reinforces why sustained monitoring, despite its associated costs, remains a cornerstone of effective prevention strategies. 

When *T. gondii* infection is confirmed during pregnancy, treatment protocols should follow a clear progression based on gestational timing and diagnostic findings [52]. Initial treatment with spiramycin is indicated until the 16th week of gestation. After this period, the therapeutic approach should be guided by amniocentesis results and ultrasonographic findings. Detection of parasitic DNA through PCR or identification of morphological changes via ultrasound suggests fetal infection, necessitating a transition to triple therapy with sulfadiazine, pyrimethamine, and folinic acid. This protocol modification considers the parasite’s complex immunological interactions, as acquired immunity is genotype-specific [53]. The development of new immunological responses is required when encountering different *T. gondii* genotypes, making sustained monitoring essential throughout pregnancy for detecting potential new or reactivated infections. This biological characteristic underscores the importance of a flexible therapeutic approach that can be adjusted based on ongoing diagnostic findings and the specific nature of the infection.

Multiple factors influence the detection of fetal infection, including infection timing, PCR sample collection timing, and treatment initiation, all of which can affect test sensitivity [42]. While imaging studies provide valuable diagnostic information, they cannot definitively rule out fetal infection, particularly retinal involvement. Our meta-analysis demonstrates that maternal treatment reduces both transmission risk (42%) and clinical manifestations (40%), with subgroup analyses indicating more consistent outcomes for combination therapy (SPF or S/SPF) compared to spiramycin alone, aligning with findings from Montoya et al. [4]. These results support transitioning from spiramycin monotherapy to combination treatment after the initial gestational period, as spiramycin alone does not effectively treat fetal infection.

The timing of the immunological response presents additional diagnostic challenges in neonatal cases. The interval between placental and fetal infection allows anti-*Toxoplasma* maternal IgG antibody transfer, which can suppress fetal antibody production. This dynamic may result in negative anti-*Toxoplasma* IgM antibodies at birth despite infection, even with highly sensitive testing methods. Conversely, when maternal infection occurs near delivery, neonates may develop positive serology within weeks after birth, though subsequent confirmatory testing may become negative. These variable serological patterns emphasize the importance of comprehensive follow-up, as demonstrated by Lago et al. [54], who found that initiating treatment within two months of life serves as a protective factor against late-onset retinochoroiditis, which may manifest later in life [55].

Neonatal treatment during the first year of life aims to control infection until the child develops a sufficient immune response to inhibit parasite proliferation. McLeod et al. [56] emphasizes that treatment requires individually compounded formulations based on the child’s weight, aligning with World Health Organization guidelines for patient safety and pharmacotherapeutic principles [56,57]. The implementation of standardized syrup formulations is particularly crucial as these medications are typically only available in adult tablet form, helping optimize dosing while minimizing handling errors and inappropriate administration. However, as Trotta et al. [33] note, even with appropriate postnatal treatment, serious complications may occur, especially in cases of first-trimester infection.

The lack of global consensus on toxoplasmosis screening during pregnancy presents an ongoing challenge [58]. While some countries mandate comprehensive maternal monitoring, others do not recommend routine screening [32,59]. Our systematic review provides evidence supporting standardized protocols that include therapeutic regimen modification after the 16th–18th weeks of pregnancy, coupled with monthly serological monitoring. This approach is particularly important given that congenital toxoplasmosis is primarily associated with delayed maternal diagnosis and subsequent treatment initiation [39]. The evidence from our analysis supports improving surveillance of women of childbearing age, pregnant women, and infected children, as such measures have demonstrated reduced incidence of congenital toxoplasmosis [38]. Additionally, our findings reinforce the importance of immediate postnatal treatment initiation and standardized medication preparation protocols [44].

Our meta-analysis presents important limitations that should be considered. The high heterogeneity observed could not be fully explained by our subgroup analyses. Important clinical factors, such as the gestational age at infection, were not consistently reported across studies, limiting our ability to control for this potential confounder. Additionally, the included studies varied in design and diagnostic criteria, contributing to the observed heterogeneity. Consequently, the aggregated results should be interpreted with caution, especially since only observational studies were included. While our findings strongly support monthly monitoring and comprehensive diagnostic approaches, we acknowledge the economic challenges faced by healthcare systems, particularly in low- and middle-income countries. Treatment approaches did not account for potential differences in gestational infection by different genotypes, which could influence treatment resistance and the immune response. Indeed, there are several studies showing that certain genotypes display differential sensitivity to common treatments that are preconized nowadays [60,61]. For instance, type I strains, known for their high virulence in mice, may exhibit resistance to some drugs due to differences in their metabolic pathways or drug target sites; type II and III strains, which are more prevalent in human infections, may show varying degrees of susceptibility based on their enzyme activity related to folate metabolism and drug detoxification; and atypical strains found in specific geographic regions may have unique genetic mutations that affect drug efficacy [60,61]. Understanding these genotype-specific responses is crucial for optimizing treatment strategies and developing more effective therapies against *T. gondii* infections. Future research should focus on developing and validating cost-effective monitoring strategies that maintain clinical efficacy while considering resource constraints in different healthcare settings. Thus, future studies on congenital toxoplasmosis need to concentrate on new diagnostic tools, novel drugs with efficacy against potentially resistant genotypes and fewer side effects, as well as innovative strategies for health education aimed at women of childbearing age.

Overall, the qualitative analysis of this systematic review focused on the main aspects defined as useful to clinical practice and that may serve as guidelines for future research. First, prior knowledge of the serological status of women of childbearing age, and the detection of seroconversion in non-reactive pregnant women or those reinfected by *T. gondii*, should be encouraged monthly. This contributes to more assertive and prompter decision-making regarding treatment and better prognosis for infections caused by this disease. Additionally, we suggest the implementation of adequate formulas specifically prepared for neonates and children to minimize improper manipulation of these medications through home preparations. This allows for the optimization of medication doses, reduces the risk of using these medications in inadequate doses, and promotes the rational use of these medications, contributing to the goal of minimizing clinical manifestations in these children.

In conclusion:

- The occurrence of congenital toxoplasmosis remains a significant health problem in numerous countries. There is no global consensus on the traceability of toxoplasmosis during pregnancy. While some countries advocate monitoring all pregnant women, others do not recommend it, and the choice of treatment conduct is not yet well-established. The frequency of congenital toxoplasmosis is primarily associated with late, inaccurate, or nonexistent diagnosis during pregnancy, leading to delays or the absence of adequate treatment;
- This systematic review and meta-analysis of articles published from 2013 to 2025, selected based on inclusion and exclusion criteria, reveals an urgent need to establish standardization for therapeutic protocols. This is particularly crucial after the 16th or 18th week of pregnancy. Additionally, monthly monitoring of pregnant women with serological tests is recommended as a predictor to reduce the vertical transmission of *Toxoplasma gondii*. Ensuring proper healthcare access and promoting adequate treatment will improve the overall health of pregnant women and their children;
- Future studies on congenital toxoplasmosis that address procedures in the mother–child dyad should concentrate on new diagnostic tools, novel drugs with efficacy against potentially resistant genotypes and fewer side effects, as well as innovative strategies for health education aimed at women of childbearing age.

## Figures and Tables

**Table 1:** General characteristics of the studies included in the meta-analysis.

Study	Country	Population (*)	Mother Treatment (Diagnosis Trimester/Week Initiated Protocol)	Newborn Treatment (*)	Conclusions
Avelino et al., 2014 [44]	Brazil	120 treated PW, 115 untreated PW, 162 treated NB, 0 untreated NB	S (2T,25W)	SPF	Treatment of pregnant women with spiramycin reduces the possibility of transmission of infection to the fetus. However, a lack of proper treatment is associated with the onset of the neural–optical form of congenital infection. Primary preventive measures should be increased for all pregnant women during the prenatal period, and secondary prophylaxis through surveillance of seroconversion in seronegative pregnant woman should be introduced to reduce the severity of congenital infection in the environment.
Conceição et al., 2021 [24]	Brazil	82 treated PW, 102 untreated PW, 29 treated NB, 0 untreated NB	Undefined (3T,29W)	SPF-C	High prevalence rates of clinical manifestations were observed in infants with congenital toxoplasmosis after a waterborne toxoplasmosis outbreak, the largest yet described. Cerebral calcifications were higher in infants with ocular abnormalities, and maternal infection during the third pregnancy trimester was associated with a higher rate of congenital toxoplasmosis independent of maternal treatment.
Damar et al., 2023 [20]	Turkey	103 treated PW, 16 untreated PW, 3 treated NB, 2 untreated NB	S (2T,15W)	C-SX	In conclusion, although *Toxoplasma* seroprevalence was found to be high in our region, there was a paucity in diagnosis, follow-up, and treatment. Our findings support that prenatal spiramycin prophylaxis is effective in preventing the transmission of parasites from mother to child.
De Santis et al., 2024 [45]	Italy	537 treated PW, 35 untreated PW, 34 treated NB, 0 untreated NB	S/SPF-Cl/S	Undefined	The study discusses the efficacy of available treatments to reduce the risk of vertical transmission of toxoplasmosis during pregnancy, highlighting the controversy over their effectiveness. Although a large randomized clinical trial would be ideal to validate or modify current clinical practices, randomization against placebo is considered unethical. The authors’ experience indicates that maternal treatment with spiramycin and cotrimoxazole, even with negative amniocentesis, can significantly reduce the rate of transmission of congenital toxoplasmosis without causing harm to the mother or fetus.
Gomes-Ferrari-Strang et al., 2023 [38]	Brazil	48 treated PW, 13 untreated PW, 61 treated NB, 0 untreated NB	S-S/SPF (2T,26W)	SPF	Based on the follow-up of women with acute *T*. *gondii* infection and their children, through a multidisciplinary team, the availability of anti-*T*. *gondii* serology and pre- and post-natal treatments reduced the risk of toxoplasmosis transmission.
Guarch-Ibáñez et al., 2024 [46]	Spain	36 treated PW, 18 untreated PW, 0 treated NB, 0 untreated NB	S-SPF-S/SPF	S/SPF	Since cases detected by prenatal screening and treated with SPI and/or PSA presented fewer complications at birth and during follow-up, it is recommended to implement universal screening in Spain and in countries with similar epidemiological data. Long-term follow-up of the REIV-TOXO cohort will provide more information on late complications and the effects of pre- and postnatal treatments.
Guegan et al., 2021 [27]	Frace/Serbia/USA	61 treated PW, 54 untreated PW, 0 treated NB, 0 untreated NB	S-SPF-S/SPF (3T,28W)	Undefined	The sensitivity of PCR for detecting *Toxoplasma* in blood was also reduced by maternal treatment from 39.1% to 23.2%. These results highlight that anti-*Toxoplasma* therapy during pregnancy may set back biological evidence of neonatal infection at birth and underline the need for a careful serological follow-up of infants with normal workup.
Lago et al., 2014 [47]	Brazil	12 treated PW, 16 untreated PW, 59 treated NB, 6 untreated NB	Undefined	Undefined	Even with high sensitivity methods, children with congenital toxoplasmosis can have a negative anti-*Toxoplasma* IgM result at birth. It is important not to interrupt the monitoring of infants with suspected congenital toxoplasmosis simply because they present a negative anti-*Toxoplasma* IgM result.
Losa et al., 2024 [48]	Portugal	63 treated PW, 7 untreated PW, 0 treated NB, 0 untreated NB	S-SPF-S/SPF	S/SPF	The lower incidence observed in the study, compared to Europe, may be related to the reduction in the prevalence of toxoplasmosis, the effectiveness of primary infection prevention measures, and a well-structured prenatal screening program, which allows early initiation of treatment to prevent vertical transmission.
Mejia-Oquendo et al., 2021 [49]	Colombia	52 treated PW, 47 untreated PW, 0 treated NB, 0 untreated NB	S-SX/P (2T,22W)	Undefined	The study showed that an early detection program for gestational toxoplasmosis implemented at a public health center in Armenia, Quindío, correctly followed evidence-based guidelines. Diagnostic tests were requested in a timely manner, with adequate follow-up of seronegative pregnant women and timely initiation of treatment. Before the implementation of the guidelines, some mothers were not treated, and their children had more ocular and neurological sequelae, something that decreased after the adoption of the recommendations. However, the frequency of infection did not decrease compared to previous studies, and there were failures in the reporting of some IgA results.
Olariu et al., 2019 [21]	USA and Romania	23 treated PW, 164 untreated PW, 0 treated NB, 0 untreated NB	Undefined	Undefined	These findings provide further evidence that anti-parasitic treatment if administered during pregnancy can contribute to better clinical outcomes, even in countries where systematic screening and treatment have not been routinely implemented.
Prusa et al., 2015 [22]	Austria	660 treated PW, 27 untreated PW, 35 treated NB, 4 untreated NB	S-SPF-S/SPF-SZ (3T,28W)	S/SPF	Amniocentesis is indicated in women with acute maternal infection and facilitated targeted therapies in pregnant women and their offspring. In women with late *T. gondii* infection, negative amniotic fluid PCR made treatment of infants unnecessary. Serological and clinical follow-up of infants is important to confirm the infection status of the infant. Recommendations, based on our 17-year experience, to improve the current diagnostic strategies and to reduce unnecessary amniocentesis, are given.
Prusa et al., 2015-2 [50]	Austria	1110 treated PW, 63 untreated PW, 141 treated NB, 0 untreated NB	S/SPF (3T,30W)	S/SPF	Results from the Austrian Toxoplasmosis Register show the efficiency of the prenatal screening program. Our results are of clinical relevance for infants, healthcare systems, and policy makers to consider preventive *T. gondii* screening as a potential tool to reduce the incidence of congenital toxoplasmosis.
Rodrigues et al., 2014 [51]	Brazil	44 treated PW, 24 untreated PW, 46 treated NB, 0 untreated NB	S (1T,13W)	SPF	The higher proportion of infants without clinical symptoms in group 1 (70.4%) suggests that maternal treatment with spiramycin delays fetal infection, reducing the clinical sequelae of the disease in newborns. Given the low sensitivity of the tests used, when there is suspicion of congenital transmission, several serological and parasitological tests are required in order to confirm or exclude congenital toxoplasmosis in newborns.
Soares et al., 2023 [30]	Brazil	46 treated PW, 33 untreated PW, 79 treated NB, 0 untreated NB	S-SPF-S/SPF (3T,28W)	SPF	A positive advance was observed regarding the care provided for the mother–child binomial affected by *T. gondii*, with a reduction in negative outcomes for the child. However, there are still challenges concerning the diagnosis and proper management of the disease.

(*) PW: pregnant women; NB: newborn; S: spiramycin; SPF: sulfadiazine + pyrimethamine + folinic acid; S/SPF: spiramycin alternate with SPF; SX: sulfadoxine; SM: sulfamethoxazol + trimetropim; AZ: azithromycin; C: corticosteroid; CL: clotrimazol.

## Data Availability

The original contributions presented in this study are included in the article/Supplementary Materials. Further inquiries can be directed to the corresponding author.

## Supplementary Materials

The following supporting information can be downloaded at: https://www.mdpi.com/article/10.3390/microorganisms13040723/s1, Table S1: Cohort selected studies among 1089 initially screened meeting inclusion but not exclusion criteria, with a focus on the treatment of congenital toxoplasmosis. Refs. [45,46,47,48,49,51,62,63,64,65,66,67,68,69,70,71,72,73,74,75,76,77,78,79,80,81] can be found in Supplementary Materials.
