# Supplementary material for: Treatment Protocols for Gestational and Congenital Toxoplasmosis: A Systematic Review and Meta-Analysis
Source: Microorganisms. 2025 Mar 24;13(4):723. doi: 10.3390/microorganisms13040723 (PMC12029831; doi:10.3390/microorganisms13040723)
Supplement: Supplementary file 1 [file microorganisms-13-00723-s001.zip › microorganisms-3497125-supplementary.pdf]

Study	Country	Numbers of patient enrolled	Maternal treatment	Newborn treatment	Major findings
**Andrade et al., 2019 [62]**	Brazil	84 treated PW, 0 untreated PW, 2 treated NB, 0 untreated NB	S- S/SPF	Undefined	The PCR method is effective in detecting toxoplasmosis and leads us to question the efficacy of maternal serological status as a diagnostic marker.
**Avci et al., 2016 [42]**	Turkey	55 treated PW, 6 untreated PW, 0 treated NB, 0 untreated NB	S	Undefined	Our results seem to encourage the use of spiramycin in women with toxoplasmosis during pregnancy.
**Avelino et al., 2014 [44]**	Brazil	120 treated PW, 115 untreated PW, 162 treated NB, 0 untreated NB	S	SPF	Treatment of pregnant women with spiramycin reduces the possibility of transmission of infection to the fetus. However, a lack of proper treatment is associated with the onset of the neural-optical form of congenital infection. Primary preventive measures should be increased for all pregnant women during the prenatal period and secondary prophylaxis through surveillance of seroconversion in seronegative pregnant woman should be introduced to reduce the severity of congenital infection in the environment.
**Bartholo et al., 2020 [41]**	Brazil	23 treated PW, 3 untreated PW, 0 treated NB, 0 untreated NB	S- S/SPF	Undefined	The rate of mother-to-child transmission of *T. gondii* is higher in untreated pregnant women and those who acquired the infection later in pregnancy.
**Boudaouara et al., 2018 [63]**	Tunisie	28 treated PW, 7 untreated PW, 35 treated NB, 0 untreated NB	S-SPF- S/SPF-SX	SPF	Thirty-four of 35 infected children were treated with pyrimethamine– sulfadiazine combination. Serological rebound was observed in seven infants. A screening program and a diagnostic algorithm in pregnant women should be implemented in Tunisia to improve the follow-up of seronegative ones and to prevent CT cases.
**Bouhlel et al., 2018 [64]**	Tunisie	26 treated PW, 1 untreated PW, 21treated NB, 0 untreated NB	S-SX	SPF	Twenty children had congenital toxoplasmosis: 19 cases were diagnosed by serology and one case was diagnosed after amniocentesis. Two children (10%) were symptomatic at birth. All children had neither clinical nor radiological signs during the follow-up.
**Buonsenso et al., 2022 [23]**	Italy	170 treated PW, 0 untreated PW, 0 treated NB, 0 untreated |NB	S-SPF-SM	SPF	The combination of Spy+TMP-SMX may be more effective in reducing the risk of maternal-fetal transmission of Toxoplasmosis compared to Spy alone; furthermore, this combination is not inferior to P/S, the current international standard-of-care maternal treatment for the prevention of CT.
**Capobiango et al., 2014 [65]**	Brazill	4 treated PW, 27 untreated PW, 29 treated NB, 2 untreated NB	S-SPF	SPF-C	The significance of the early diagnosis and treatment of toxoplasmosis during pregnancy to reduce congenital toxoplasmosis and its consequences.
**Carellos et al., 2017 [66]**	Brazil	10 treated PW, 161 untreated PW, 170 treated NB, 8 untreated NB	S	SPF	With adequate monitoring, antiparasitic treatment was feasible and relatively safe in the setting of this large screening program for congenital toxoplasmosis.
**Caro-Garzón et al., 2021 [67]**	Colombia	16 treated PW, 6 untreated PW, 22 treated NB, 0 untreated NB	Undefined	SPF	En esta cohorte de niños colombianos con toxoplasmosis congénita se encontraron los mismos resultados reportados en series previas con un mayor número de casos en Italia. La avidez no mostró ser útil para el seguimiento de los niños con toxoplasmosis congénita.
**Carral et al., 2013 [19]**	Argentina	35 treated PW, 2 untreated PW, 2 treated NB, 0 untreated NB	S/SPF	S-C	The transmission rates obtained allow consider this prevention program as a valid resource to minimize the impact of congenital toxoplasmosis.
**Conceição et al., 2021 [24]**	Brazil	82 treated PW, 102 untreated PW, 29 treated NB, 0 untreated |NB	Undefined	SPF-C	High prevalence rates of clinical manifestations were observed in infants with congenital toxoplasmosis after a waterborne toxoplasmosis outbreak, the largest yet described.
**Damar et al., 2023 [20]**	Turkey	103 treated PW, 16 untreated PW, 3 treated NB, 2 untreated |NB	S	C-SX	In conclusion, although *Toxoplasma* seroprevalence was found to be high in our region, there was a paucity in diagnosis, follow-up, and treatment. Our findings support that prenatal spiramycin prophylaxis is effective in preventing the transmission of parasites from mother to child.
**De Araújo et al., 2021 [25]**	Brazil	11 treated PW, 179 untreated PW, 190 treated NB, 0 untreated NB	S-SPF	SPF-C	Thus, although the therapeutic intervention guided the clinical outcome towards a cicatricial status of retinochoroidal lesions, the implementation of an active prenatal screening program would be the assurance that CT transmission would be prevented, avoiding the long-term impact of this infection on the infant quality of life.
**De Paula et al., 2022 [68]**	Brazil	42 treated PW, 0 untreated PW, 3 treated NB, 0 untreated NB	S-SPF- S/SPF	SPF	Three children had the congenital form of the infection, and of these, only 1 had a positive IgM result. The serological response detected at the time of diagnosis of the infection is heterogeneous, which can make it difficult to interpret the tests, due to the presence of non-classical serological profiles.

**De Santis et al., 2024 [45]**	Italy	537 treated PW, 35 untreated PW, 34 treated NB, 0 untreated NB	S/SPF-Clotrimazol/Espiramicina	Undefined	The study discusses the efficacy of available treatments to reduce the risk of vertical transmission of toxoplasmosis during pregnancy, highlighting the controversy over their effectiveness. Although a large randomized clinical trial would be ideal to validate or modify current clinical practices, randomization against placebo is considered unethical. The authors' experience indicates that maternal treatment with Spiramycin and Cotrimoxazole, even with negative amniocentesis, can significantly reduce the rate of transmission of congenital toxoplasmosis without causing harm to the mother or fetus.
**Diesel et al., 2019 [69]**	Brazil	65 treated PW, 0 untreated PW, 4 treated NB, 2 untreated NB	S-SPF- S/SPF-AZ	SPF	The present study suggests that research on PCR screening of the amniotic fluid may be useful to identify patients with a higher potential for fetal complications, who may benefit from the poly-antimicrobial treatment. Patients with negative PCR results must continue to prevent fetal infection with monotherapy, without risk of fetal or maternal impairment.
**Donadono et al., 2019 [70]**	Italy	564 treated PW, 0 untreated PW, 0 treated NB, 0 untreated NB	S-SPF- S/SPF	Undefined	Toxoplasmosis is uncommon in pregnancy with overall incidence of seroconversion and suspected infection in of women referred to our center for suspected infection were considered not infected
**Donadono et al., 2022 [71]**	Italy	218 treated PW, 0 untreated PW, 0 treated NB, 0 untreated NB	S-SPF- S/SPF	Undefined	In conclusion, there is a positive association between L+ status in pregnant women, and risk of CT also confirmed when restricting the analysis to women with early diagnosis of seroconversion and treatment. This data could be very useful in counselling pregnant women with toxoplasmosis seroconversion and lead to direct a more specific therapeutic and diagnostic protocol.
**Evangelista et al., 2020 [72]**	Brazil	218 treated PW, 0 untreated PW, 0 treated NB, 0 untreated NB	S- S/SPF	Undefined	The monthly serology of non-reactive pregnant women to *T. gondii* is a fundamental control mechanism, especially in developing countries with a high prevalence of toxoplasmosis and genotypic diversity of the parasite.
**Findal et al., 2017 [26]**	Turkey	302 treated PW, 4 untreated PW, 0 treated NB, 0 untreated NB	S-SPF- S/SPF-AZ	Undefined	The inclusion of *Toxoplasma* in routine screening program is still controversial and differs between countries. If infection is detected, treatment may be recommended because it may reduce the transmission to the fetus
**Fricker-Hidalgo et al., 2013 [34]**	France	22 treated PW, 4 untreated PW, 0 treated NB, 0 untreated NB	S	Undefined	When the interpretation of serological results is so difficult, it seems careful to initiate treatment by spiramycin and to follow the pregnant women and their children.
**Gomes-Ferrari-Strang et al., 2023 [38]**	Brazil	48 treated PW, 13 untreated PW, 61 treated NB, 0 untreated NB	S- S/SPF	SPF	The follow-up of women with acute *T. gondii* infection and their children, through a multidisciplinary team, availability of anti-*T. gondii* serology and pre- and post-natal treatments reduced the risk of toxoplasmosis transmission.
**Guarch-Ibáñez et al., 2024 [46]**	Espanha	36 treated PW, 18 untreated PW, 0 treated NB, 0 untreated NB	S-SPF-S/SPF	Undefined	Since cases detected by prenatal screening and treated with SPI and/or PSA presented fewer complications at birth and during follow-up, it is recommended to implement universal screening in Spain and in countries with similar epidemiological data. Long-term follow-up of the REIV-TOXO cohort will provide more information on late complications and the effects of pre- and postnatal treatments.
**Guegan et al., 2021 [27]**	France, Serbia and USA	61 treated PW, 54 untreated PW, 0 treated NB, 0 untreated NB	S-SPF- S/SPF	Undefined	The sensitivity of *Toxoplasma* PCR in blood was also lowered by maternal treatment from 39.1% to 23.2%. These results highlight that anti-*Toxoplasma* therapy during pregnancy may set back biological evidence of neonatal infection at birth and underline the need for a careful serological follow-up of infants with normal workup.
**Hijikata et al., 2022 [37]**	Japan	52 treated PW, 19 untreated PW, 0 treated NB, 0 untreated NB	S	Undefined	A few children born to anti-*T. gondii* IgM-positive mothers were suspected of having congenital T. gondii infection based on serum anti-*T. gondii* IgM or blood *T. gondii* DNA testing at birth. However, none developed congenital *T. gondii* infection.
**Kalem et al., 2022 [59]**	Turkey	11 treated PW, 13 untreated PW, 0 treated NB, 0 untreated NB	S	Undefined	Anti-*T. gondii* IgM positivity is an indication of acute infection. But IgM can persist for years and be false-positive in pregnancy. Therefore, additional tests are required and leading to emotional distress and unnecessary interventions in pregnancy women. These results can aid in developing an approach to screening and diagnosis of *T. gondii* infection in pregnancy.
**Kamus et al., 2023 [3]**	France	16 treated PW, 33 untreated PW, 10 treated NB, 6 untreated NB	Undefined	Undefined	In conclusion, although *Toxoplasma* seroprevalence was found to be high in our region, there was a paucity in diagnosis, follow-up, and treatment. Our findings support that prenatal spiramycin prophylaxis is effective in preventing the transmission of parasites from mother to child. information to physicians and the population, to improve management and epidemiological monitoring.

**Koçak and Kan, 2020 [58]**	Turkey	7 treated PW, 0 untreated PW, 0 treated NB, 0 untreated NB	S	Undefined	The inclusion of *Toxoplasma* in routine screening program is still controversial and differs between countries. Screening in areas with a high rate of T*oxoplasma*, such as in our country, may be rational. If infection is detected, treatment may be recommended because it may reduce the transmission to the fetus.
**Lago et al., 2014 [47]**	Brazil	12 treated PW, 16 untreated PW, 59 treated NB, 6 untreated NB	Undefined	Undefined	Even with high sensitivity methods, children with congenital toxoplasmosis can have negative anti-*Toxoplasma* IgM result at birth. It is important not to interrupt the monitoring of infants with suspected congenital toxoplasmosis simply because they present a negative anti-*Toxoplasma* IgM result.
**Lago et al., 2021 [54]**	Brazil	20 treated PW, 57 untreated PW, 73 treated NB, 4 untreated |NB	S-SPF	SPF-C	The high incidence of new retinochoroidal lesions during the follow-up period indicates the importance of long-term follow-up of patients with congenital toxoplasmosis. Initiating treatment within the first 4 months of life, especially within the first 2 months, was a protective factor against the later development of retinochoroiditis.
**Losa et al., 2024 [48]**	Portugal	63 treated PW, 7untreated PW, 0 treated NB, 0 untreated NB	S-SPF-S/SPF	Undefined	The lower incidence observed in the study, compared to Europe, may be related to the reduction in the prevalence of toxoplasmosis, the effectiveness of primary infection prevention measures and a well-structured prenatal screening program, which allows early initiation of treatment to prevent vertical transmission.
**Ludwig et al., 2022 [73]**	Brazil	40 treated PW, 0 untreated PW, 2 treated NB, 0 untreated NB	S-SPF- S/SPF-AZ	SPF	When correlating the treatment time and the detection of DNA in the placentas, no significant result was found. The prevalence of positive samples was lower than in other studies in the literature. The data reaffirm the importance of carrying out the analysis of the placenta.
**Mandelbrot et al., 2018 [43]**	France	143 treated PW, 0 untreated PW, 0 treated NB, 0 untreated NB	S-SPF	Undefined	There was a trend toward lower transmission with pyrimethamine þ sulfadiazine, but it did not reach statistical significance, possibly for lack of statistical power because enrollment was discontinued. There were also no fetal cerebral toxoplasmosis lesions in the pyrimethamine þ sulfadiazine group.
**Mejia-Oquendo et al., 2021 [49]**	Colombia	52 treated PW, 47 untreated PW, 0 treated NB, 0 untreated NB	S-Sulfadoxina/P	Undefined	The study showed that an early detection program for gestational toxoplasmosis implemented at a public health center in Armenia, Quindío, correctly followed evidence-based guidelines. Diagnostic tests were requested in a timely manner, with adequate follow-up of seronegative pregnant women and timely initiation of treatment. Before the implementation of the guidelines, some mothers were not treated, and their children had more ocular and neurological sequelae, something that decreased after the adoption of the recommendations. However, the frequency of infection did not decrease compared to previous studies, and there were failures in the reporting of some IgA results.
**Mueller et al., 2021 [74]**	Brazil	15 treated PW, 29 untreated PW, 10 treated NB, 36 untreated NB	S-SPF- S/SPF	Undefined	Despite the existence of national recommendations, several opportunities were missed to prevent CT during the antenatal period and to diagnose and treat this condition in the neonatal period.
**Pengsaa and Hattasingh, 2015 [75]**	Thailand	0 treated PW, 0 untreated PW, 2 treated NB, 18 untreated NB	Undefined	SPF-S/SPF-C	Twenty cases of congenital toxoplasmosis are reported. Delayed diagnosis and treatment resulted in a poor outcome. The prevention of toxoplasmosis in pregnant women and prompt diagnosis and appropriate treatment of congenital toxoplasmosis should be a priority to prevent a poor outcome in infected children.
**Piffer et al., 2020 [28]**	Italy	123 treated PW, 0 untreated PW, 0 treated NB, 0 untreated NB	S- S/SPF	Undefined	The extent of serological screening and the high treatment rate helped to keep the risk of infection transmission to the foetus low and to achieve a very low rate of congenital infection
**Prasil et al., 2023 [76]**	Czech Republic	122 treated PW, 0 untreated PW, 0 treated NB, 0 untreated NB	S-SPF- S/SPF	Undefined	The superiority of one of the therapeutic regimens was not statistically demonstrated, since the differences in overall toxicity or incidence of toxic allergic reactions between the cohorts were not confirmed (p¼.53 and p¼1.00, respectively). However, although the isolated neurotoxicity of spiramycin was the only significant adverse reaction demonstrated in this study, pyrimethamine/sulfadiazine therapy should be preferred, because it is known to be more effective and with limited adverse reactions
**Prusa et al., 2015 [22]**	Austria	660 treated PW, 27 untreated PW, 35 treated NB, 4 untreated |NB	S-SPF-S/SPF- SX	S/SPF	Amniocentesis is indicated in women with acute maternal infection and facilitated targeted therapies in pregnant women and their offspring. In women with late *Toxoplasma* infection, negative amniotic fluid PCR made treatment of infants unnecessary. Serological and clinical follow-up of infants is important to confirm the infection status of the infant. Recommendations, based on our 17-year experience, to improve the current diagnostic strategies and to reduce unnecessary amniocentesis, are given.

**Prusa et al., 2015-2 [50]**	Austria	1110 treated PW, 63 untreated PW, 141 treated NB, 0 untreated NB	S/SPF	S/SPF	Results from the Austrian Toxoplasmosis Register show the efficiency of the prenatal screening program. Our results are of clinical relevance for infants, healthcare systems, and policy makers to consider preventive *Toxoplasma* screening as a potential tool to reduce the incidence of congenital toxoplasmosis
**Righi et al., 2021 [39]**	Brazil	58 treated PW, 16 untreated PW, 0 treated NB, 0 untreated NB	S-SPF- S/SPF	Undefined	The occurrence of congenital toxoplasmosis is associated with late diagnosis, in the last trimester of pregnancy, and consequent lack of adequate treatment. In addition, there was a high prevalence of children infected by vertical transmission due to the outbreak, which reinforces the importance of prenatal care and attention to the need for adequate treatment and care during the development of infected children.
**Rodrigues et al., 2014 [51]**	Brazil	44 treated PW, 24 untreated PW, 46 treated NB, 0 untreated |NB	S	SPF	The higher proportion of infants without clinical symptoms in group 1 (70.4%) suggests the maternal treatment with spiramycin delays fetal infection, reducing the clinical sequelae of the disease in children. Given the low sensitivity of the tests used, when there is suspicion of congenital transmission, several serological and parasitological tests are required to confirm or exclude congenital toxoplasmosis in children.
**Saghrouni et al., 2013 [77]**	Tunisie	16 treated PW, 5 untreated PW, 20 treated NB, 1 untreated NB	S-S/SPF	Undefined	Severe congenital toxoplasmosis is still present in our country. The care of pregnant women at risk needs to be improved.
**Soares et al., 2023 [30]**	Brazil	46 treated PW, 33 untreated PW, 79 treated |NB, 0 untreated NB	S-SPF- S/SPF	SPF	A positive advance was observed regarding the care provided for the mother-child binomial affected by *T. gondii*, with a reduction in negative outcomes for the child. However, there are still challenges concerning the diagnosis and proper management of the disease.
**Olariu et al., 2019 [21[**	USA and Romania	23 treated PW, 164 untreated PW, 0 treated NB, 0 untreated NB	Undefined	Undefined	These findings provide further evidence that anti-parasitic treatment if administered during pregnancy can contribute to better clinical outcomes, even in countries where systematic screening and treatment have not been routinely implemented.
**Teil et al., 2016 [78]**	France	44 treated PW, 21 untreated PW, 65 treated NB, 0 untreated NB	Undefined	SPF	According to our results and previously published data, the combination of sulfadoxine–pyrimethamine seems to be well tolerated. However, the sample size of our study was too small to rule out the risk of less frequent, but nevertheless severe, reactions and, in particular, of hypersensitivity reactions.
**Trotta et al., 2021 [33]**	Italy	1 treated PW, 0 untreated PW, 1 treated NB, 0 untreated NB	S- S/SPF	SPF	Toxoplasmosis acquired in early pregnancy has a low risk of fetal infection. Actively discussing case-by-case amniocentesis indication with patients, especially when a recent toxoplasmosis is not properly confirmed, is desirable.
**Valentini et al., 2015 [31]**	Italy	123 treated PW, 0 untreated PW, 123 treated NB, 0 untreated NB	S-SPF-SM	Undefined	The treatment based on Sp/C has significant efficacy in reducing maternal-fetal transmission of *Toxoplasma gondii* when compared with Pyr/Sul and particularly to Spy. Randomized controlled trials would be required.
**Villar et al., 2020 [79]**	Brazil	163 treated PW, 0 untreated PW, 0 treated NB, 0 untreated NB	S-SPF	Undefined	Late referral to specialized medical services, inadequate toxoplasmosis management at the original prenatal care services, and social vulnerabilities are contributing factors to the persistent occurrence of congenital toxoplasmosis cases.
**Vimercati et al., 2020 [35]**	Italy	325 treated PW, 0 untreated PW, 0 treated NB, 0 untreated NB	S- S/SPF	Undefined	Results obtained in the present study suggest that the new IgG avidity-based classification herein proposed could estimate more precisely the likelihood of a primary maternal *Toxoplasma* infection as well as the risk of fetal infection, when compared with the historical Lebech Classification.
**Wallon et al., 2013 [36]**	France	1901 treated PW, 139 untreated PW, 485 treated NB, 28 untreated NB	S-S/SPF	SPF	These analyses demonstrated that introduction of monthly prenatal screening and improvement in antenatal diagnosis were associated with a significant reduction in the rate of congenital infection and a better outcome at 3 years of age in infected children.
**Wallon et al., 2014 [55]**	France	389 treated PW, 88 untreated PW, 470 treated NB, 7 untreated NB	S-SPF-S/SPF	SPF-SX	Although the consequences of CT are rarely severe in treated children, regular postnatal monitoring is nevertheless justified because of the lifelong persisting risk of new ocular manifestations.
**Yamada et al., 2019 [32]**	Japan	6 treated PW, 1 untreated PW, 1 treated NB, 6 untreated NB	S/SPF	SPF	Seven of the 12 cases were diagnosed as having congenital *T. gondii* infection, and they had low IgG avidity indices. Congenital *T. gondii* infection screening using of IgG avidity and multiplex-nested PCR methods for pregnant women with a positive test for *T. gondii* antibody plus a positive or equivocal test for *T. gondii* IgM was useful for detecting a high-risk pregnancy and diagnosing congenital *T. gondii* infection.

**Yamamoto et al., 2017 [80]**	Brazil	122 treated PW, 0 untreated PW, 122 treated NB, 0 untreated NB	S	SPF-C	Parasite load in AF is associated with the clinical outcome in congenital toxoplasmosis, irrespective of gestational age at maternal infection
**Zuluaga et al., 2017 [81]**	Colombia	15 treated PW, 8 untreated PW, 0 treated NB, 0 untreated NB	S	Undefined	These results show that treatment with spiramycin during pregnancy in gestational toxoplasmosis reduced the relative risk of developing the disease in the children by 96% (95% CI 33-100%).

Supplementary Table S1. Cohort selected studies among 1,089 initially screened meeting inclusion but not exclusion criteria, with a focus on the treatment of congenital toxoplasmosis. PW: pregnancy women; NB: newborn; S: spiramycin; SPF: sulfadiazine + pyrimethamine + folinic acid; S/SPF: spiramycin alternate with SPF; SPF: sulfadiazine + pyrimethamine + folinic acid; SX: sulfadoxine; SM: sulfamethoxazole + trimetropim; AZ: azithromycin; C: corticosteroid.

REFERENCES

62. ANDRADE, F. M. *et al.* Polymerase chain reaction analysis of amniotic fluid for diagnosis of fetal toxoplasmosis. **Clinical and Experimental Obstetrics and Gynecology**, 2019, 46(4), 593–595. doi.org/10.12891/ceog4585.2019.

42. AVCI, M. E. *et al.* Role of spiramycin in prevention of fetal toxoplasmosis. The journal of maternal-fetal & neonatal medicine : the official journal of the European Association of Perinatal Medicine, the Federation of Asia and Oceania Perinatal Societies, **Journal of Maternal-Fetal & Neonatal Medicine**, England. 2016;29(13):2073-6. doi: 10.3109/14767058.2015.1074998.

44. AVELINO, M. M. *et al.* Congenital toxoplasmosis and prenatal care state programs. **BMC Infectious Diseases**. 2014 Jan 18;14:33. doi: 10.1186/1471-2334-14-33.

41. BARTHOLO, B. B.G.R. et al. Treatment of acute toxoplasmosis in pregnancy: influence in the mother-to-child transmission. **Journal of Obstetrics and Gynaecology Canada**, 2020 Dec;42(12):1505-1510. doi: 10.1016/j.jogc.2020.04.021.

63. BOUDAOUARA, Y. *et al.* Congenital toxoplasmosis in Tunisia: Prenatal and neonatal diagnosis and postnatal follow-up of 35 cases. **American Journal of Tropical Medicine and Hygiene**, 2018 Jun;98(6):1722-1726. doi: 10.4269/ajtmh.17-0580.

64. BOUHLEL, S. *et al.* [Management of Toxoplasmic Seroconversion in the Third Trimester of Pregnancy in Tunisia]. **Bulletin de la Societe de Pathologie Exotique (1990)**, France, 2018;111(5):269-274. French. doi: 10.3166/bspe-2019-0054..

23. BUONSENSO, D. *et al.* Spyramicine and Trimethoprim-Sulfamethoxazole Combination to Prevent Mother-To-Fetus Transmission of *Toxoplasma gondii* Infection in Pregnant Women: A 28-Years Single-center Experience. **Pediatric Infectious Disease Journal**, 2022 May 1;41(5):e223-e227. doi: 10.1097/INF.0000000000003469.

65. CAPOBIANGO, J.D. *et al.* Congenital toxoplasmosis in a reference center of Paraná, Southern Brazil. **The Brazilian Journal of Infectious Diseases**, Brazil, 2014 Jul-Aug;18(4):364-71. doi: 10.1016/j.bjid.2013.11.009.

66. CARELLOS, E. V. M. *et al.* High Frequency of Bone Marrow Depression During Congenital Toxoplasmosis Therapy in a Cohort of Children Identified By Neonatal Screening in Minas Gerais, Brazil. **Pediatric Infectious Disease Journal**, 2017 Dec;36(12):1169-1176. doi: 10.1097/INF.0000000000001561.

67. CARO-GARZÓN, J. D. *et al.* Evaluation of the avidity test for the follow up on children treated for congenital toxoplasmosis during the first year of life. **Iatreia**, 2021, vol.34, n.1, pp.25-32. ISSN 0121-0793. https://doi.org/10.17533/udea.iatreia.70.

19. CARRAL, L. *et al.* Prevention of congenital toxoplasmosis in a Buenos Aires hospital. **Medicina**, Argentina, 2013;73(3):238-42. Spanish. PMID: 23732199.

24. CONCEIÇÃO, A. R. *et al.* Ocular Findings in Infants with Congenital Toxoplasmosis after a Toxoplasmosis Outbreak. **Ophthalmology**, 2021 Sep;128(9):1346-1355. doi: 10.1016/j.ophtha.2021.03.009.

20. DAMAR ÇAKIRCA, T. *et al.* Toxoplasmosis: A Timeless Challenge for Pregnancy. **Tropical Medicine**

and Infectious Disease, 2023 Jan 13;8(1):63. doi: 10.3390/tropicalmed8010063.

25. DE ARAÚJO, T. E. *et al.* Long-term impact of congenital toxoplasmosis on phenotypic and functional features of circulating leukocytes from infants one year after treatment onset. **Clinical Immunology**, 2021 Nov;232:108859. doi: 10.1016/j.clim.2021.108859.

68. DE PAULA, H. L. *et al.* Delta-aminolevulinate dehydratase enzyme activity and the oxidative profile of pregnant women being treated for acute toxoplasmosis. **Microbial Pathogenesis**, 2022 Mar;164:105455. doi: 10.1016/j.micpath.2022.105455.

45. DE SANTIS, M. *et al.* The prevention of congenital toxoplasmosis using a combination of Spiramycin and Cotrimoxazole: The long-time experience of a tertiary referral centre. **Tropical Medicine & International Health**, 2024 Aug;29(8):697-705. doi: 10.1111/tmi.14021.

69. DIESEL, A. A. *et al.* Follow-up of Toxoplasmosis during Pregnancy: Ten-Year Experience in a University Hospital in Southern Brazil. **Revista Brasileira de Ginecologia e Obstetricia**, 41 (09) • Sept 2019 • https://doi.org/10.1055/s-0039-1697034.

70. DONADONO, V. *et al.* Incidence of toxoplasmosis in pregnancy in Campania: A population-based study on screening, treatment, and outcome. **European Journal of Obstetrics, Gynecology, and Reproductive Biology**, Ireland, 2019 Sep;240:316-321. doi: 10.1016/j.ejogrb.2019.07.033.

71. DONADONO, V. *et al.* Association between lymphadenopathy after toxoplasmosis seroconversion in pregnancy and risk of congenital infection. **European Journal of Clinical Microbiology & Infectious Diseases**, Germany, 2022 Jan;41(1):45-51. doi: 10.1007/s10096-021-04337-9.

72. EVANGELISTA, F. F. *et al.* Prospective evalution of pregnant women with suspected acute toxoplasmosis treated in a reference prenatal care clinic at a university teaching hospital in Southern Brazil. **Revista do Instituto de Medicina Tropical de Sao Paulo**, Brazil, 62 • 2020 • https://doi.org/10.1590/S1678-9946202062046.

26. FINDAL, G. *et al.* Management of suspected primary *Toxoplasma gondii* infection in pregnant women in Norway: Twenty years of experience of amniocentesis in a low-prevalence population. **BMC Pregnancy and Childbirth**, 2017 Apr 26;17(1):127. doi: 10.1186/s12884-017-1300-1.

34. FRICKER-HIDALGO, H. *et al. Toxoplasma* seroconversion with negative or transient immunoglobulin M in pregnant women: Myth or reality? A French multicenter retrospective study. **Journal of Clinical Microbiology**, 2013 Jul;51(7):2103-11. doi: 10.1128/JCM.00169-13.

38. GOMES-FERRARI-STRANG, A. G. *et al*. Gestational toxoplasmosis treatment changes the child's prognosis: A cohort study in southern Brazil. **PLoS Neglected Tropical Diseases**, 2023 Sep 29;17(9):e0011544. doi: 10.1371/journal.pntd.0011544.

46. GUARCH-IBÁÑEZ, B. *et al.* REIV-TOXO Project: Results from a Spanish cohort of congenital toxoplasmosis (2015–2022). The beneficial effects of prenatal treatment on clinical outcomes of infected newborns. **PLOS Neglected Tropical Diseases**, 2024 Oct 22;18(10):e0012619. doi: 10.1371/journal.pntd.0012619..

27. GUEGAN, H. *et al.* Maternal Anti-*Toxoplasma* Treatment during Pregnancy Is Associated with Reduced Sensitivity of Diagnostic Tests for Congenital Infection in the Neonate. **Journal of Clinical Microbiology**, United States, 2021 Jan 21;59(2):e01368-20. doi: 10.1128/JCM.01368-20.

37. HIJIKATA, M. *et al.* A prospective cohort study of newborns born to mothers with serum *Toxoplasma gondii* immunoglobulin M positivity during pregnancy. **Journal of Infection and Chemotherapy**, 2022 Apr;28(4):486-491. doi: 10.1016/j.jiac.2021.12.005.

3. KAMUS, L. *et al.* Maternal and congenital toxoplasmosis in Mayotte: Prevalence, incidence and management. **Plos Neglected Tropical Diseases**, 2023 Mar 20;17(3):e0011198. doi: 10.1371/journal.pntd.0011198.

59. KALEM, A. K. *et al.* Toxoplasmosis in pregnancy: test, treatment and outcome. **The European Research Journal**, 2022;8(2):296-303, doi: 10.18621/eurj.1039212

58. KOÇAK, Ö., KAN, Ö. Results of the toxoplasmosis screening in 9311 pregnant women in a tertiary center in Turkey. **Flora**, 2020;25(3):332-338, doi: 10.5578/flora.69026.

47. LAGO, E. G. *et al*. Presence and duration of anti-*Toxoplasma gondii* immunoglobulin M in infants with congenital toxoplasmosis. **Jornal de Pediatria**, 2014 Jul-Aug;90(4):363-9. doi: 10.1016/j.jped.2013.12.006.

54. LAGO, E. G. *et al.* Ocular Outcome of Brazilian Patients With Congenital Toxoplasmosis. **The Pediatric Infectious Disease Journal**, United States, United States, 2021 Jan;40(1):e21-e27. doi: 10.1097/INF.0000000000002931.

48. LOSA, A. *et al.* Congenital Toxoplasmosis Diagnosis: Challenges and Management Outcomes. **Cureus**, 2024 Jan 26;16(1):e52971. doi: 10.7759/cureus.52971.

73. LUDWIG, A. *et al.* Molecular detection of *Toxoplasma gondii* in placentas of women who received therapy during gestation in a toxoplasmosis outbreak. **Infection, Genetics and Evolution**, Netherlands, 2022 Jan;97:105145. doi: 10.1016/j.meegid.2021.105145.

43. MANDELBROT, L. *et al.* Prenatal therapy with pyrimethamine plus sulfadiazine vs spiramycin to reduce placental transmission of toxoplasmosis: a multicenter, randomized trial. **American Journal of Obstetrics and Gynecology**, 2018 Oct;219(4):386.e1-386.e9. doi: 10.1016/j.ajog.2018.05.031.

49. MEJIA-OQUENDO, M. *et al*. Evaluation of the impact of the first evidence-based guidelines for congenital toxoplasmosis in Armenia (Quindío) Colombia: An observational retrospective analysis. **The Lancet Regional Health - Americas**, 2021 Jul 13;1:100010. doi: 10.1016/j.lana.2021.100010.

74. MUELLER, R. A. S. *et al.* Congenital Toxoplasmosis: Missed Opportunities for Diagnosis and Prevention. **Journal of Tropical Pediatrics**, England, 2021 Jan 29;67(1):fmaa069. doi: 10.1093/tropej/fmaa069.

21. OLARIU, T. R. *et al.* Congenital toxoplasmosis in the United States: clinical and serologic findings in infants born to mothers treated during pregnancy. **Parasite**, France, 2019;26:13. doi: 10.1051/parasite/2019013.

75. PENGSAA, K., HATTASINGH, W. Congenital toxoplasmosis: an uncommon disease in Thailand. **Paediatrics and International Child Health**, England, 2015 Feb;35(1):56-60. doi: 10.1179/2046905514Y.0000000149.

28. PIFFER, S. *et al. Toxoplasma gondii* infection during pregnancy: a ten-year observation in the province of Trento, Italy. **Le Infezioni in Medicina**, Italy, 2020 Nov 1;28(4):603-610. PMID: 33257637.

76. PRASIL, P. *et al.* Comparison of adverse reactions of spiramycin versus pyrimethamine/sulfadiazine treatment of toxoplasmosis in pregnancy: is spiramycin really the drug of choice for unproven infection of the fetus?. **Journal of Maternal-fetal & Neonatal Medicine**, England, 2023 Dec;36(1):2215377. doi: 10.1080/14767058.2023.2215377.

22. PRUSA, A. R. *et al.* Amniocentesis for the detection of congenital toxoplasmosis: results from the nationwide Austrian prenatal screening program. **Clinical Microbiology and Infection**, England, 2015 Feb;21(2):191.e1-8. doi: 10.1016/j.cmi.2014.09.018.

50. PRUSA, A. R. *et al.* The Austrian Toxoplasmosis Register, 1992-2008. **Clinical Infectious Diseases**, United States, 2015-2 Jan 15;60(2):e4-e10. doi: 10.1093/cid/ciu724.

39.RIGHI, N. C. *et al.* Epidemiological profile of gestational and congenital toxoplasmosis cases arising out of the population outbreak. **Scientia Medica**, 2021 Jan-Dec; 31: e1-e7, doi: 10.15448/1980-6108.2021.1.40108.

51. RODRIGUES, I.M. *et al.* Assessment of laboratory methods used in the diagnosis of congenital toxoplasmosis after maternal treatment with spiramycin in pregnancy. **BMC Infectious Diseases**, 2014 Jun 24;14:349. doi: 10.1186/1471-2334-14-349.

77. SAGHROUNI, F. *et al.* La toxoplasmose congénitale : à propos de 21 cas. **Journal de Pédiatrie et de Puériculture**, 2013 April; 26(2):83–89. doi: 10.1016/j.jpp.2013.01.004.

30. SOARES, J. A. S. *et al.* Profile of pregnant women and children accompanied due to *T. gondii* exposure at a referred healthcare center: What has changed in 10 years?. **Revista Brasileira de Saude Materno Infantil**, 2023 March; 23. doi: 10.1590/1806-9304202300000225.

78. TEIL, J. *et al.* Treatment of Congenital Toxoplasmosis: Safety of the Sulfadoxine-Pyrimethamine Combination in Children Based on a Method of Causality Assessment. **The Pediatric Infectious Disease Journal**, United States, 2016 Jun;35(6):634-8. doi: 10.1097/INF.0000000000001120.

33. TROTTA, M. *et al.* Primary toxoplasmosis acquired during early pregnancy: Is it currently overestimated?. **European Journal of Obstetrics and Gynecology and Reproductive Biology**, 2021 Dec;267:285-289. doi: 10.1016/j.ejogrb.2021.11.019.

31. VALENTINI, P. *et al.* Spiramycin/cotrimoxazole versus pyrimethamine/sulfonamide and spiramycin alone for the treatment of toxoplasmosis in pregnancy. **Journal of Perinatology**, 2015 Feb;35(2):90-4. doi: 10.1038/jp.2014.161.

79. VILLAR, B. B, D. L. F. *et al.* Toxoplasmosis in pregnancy: a clinical, diagnostic, and epidemiological study in a referral hospital in Rio de Janeiro, Brazil. **The Brazilian Journal of Infectious Diseases**, Brazil, 2020 Nov-Dec;24(6):517-523. doi: 10.1016/j.bjid.2020.10.001.

35. VIMERCATI, A. *et al.* Congenital toxoplasmosis and proposal of a new classification for the likelihood of primary maternal infection: analysis of 375 cases in Southeast Italy. **Journal of Maternal-Fetal and Neonatal Medicine**, 2020 Nov;33(22):3746-3751. doi: 10.1080/14767058.2019.1583737.

36. WALLON, M. *et al.* Congenital toxoplasma infection: Monthly prenatal screening decreases transmission rate and improves clinical outcome at age 3 years. **Clinical Infectious Diseases**, 2013, 56, 1223–1231. https://doi.org/10.1093/cid/cit032.

55. WALLON, M. *et al*. Ophthalmic outcomes of congenital toxoplasmosis followed until adolescence. **Pediatrics** 2014, 133, e601–e608. https://doi.org/10.1542/peds.2013-2153.

32. YAMADA, H. *et al.* A cohort study of maternal screening for congenital *Toxoplasma gondii* infection: 12 years' experience. **Journal of Infection and Chemotherapy**, 2019 Jun;25(6):427-430. doi: 10.1016/j.jiac.2019.01.009.

80. YAMAMOTO, L. *et al.* Association of Parasite Load Levels in Amniotic Fluid With Clinical Outcome in Congenital Toxoplasmosis. **Obstetrics and Gynecology**, 2017 Aug;130(2):335-345. doi: 10.1097/AOG.0000000000002131.

81. ZULUAGA, L. M. *et al.* Effect of antenatal spiramycin treatment on the frequency of retinochoroiditis due to congenital toxoplasmosis in a Colombian cohort. **Biomedica**, 2017, vol.37, suppl.1, pp.86-91. ISSN 0120-4157. doi: 10.7705/biomedica.v37i2.2818.
